# Complete mitochondrial genomes of two flat-backed millipedes by next-generation sequencing (Diplopoda, Polydesmida)

**DOI:** 10.3897/zookeys.637.9909

**Published:** 2016-11-28

**Authors:** Yan Dong, Lixin Zhu, Yu Bai, Yongyue Ou, Changbao Wang

**Affiliations:** 1 College of Biology and Food Engineering, Chuzhou University, Chuzhou 239000, China

**Keywords:** gene order, mitochondrial genome, Myriapoda, next-generation sequence, Polydesmida

## Abstract

A lack of mitochondrial genome data from myriapods is hampering progress across genetic, systematic, phylogenetic and evolutionary studies. Here, the complete mitochondrial genomes of two millipedes, *Asiomorpha coarctata* Saussure, 1860 (Diplopoda: Polydesmida: Paradoxosomatidae) and *Xystodesmus* sp. (Diplopoda: Polydesmida: Xystodesmidae) were assembled with high coverage using Illumina sequencing data. The mitochondrial genomes of the two newly sequenced species are circular molecules of 15,644 bp and 15,791 bp, within which the typical mitochondrial genome complement of 13 protein-coding genes, 22 tRNAs and two ribosomal RNA genes could be identified. The mitochondrial genome of *Asiomorpha coarctata* is the first complete sequence in the family Paradoxosomatidae (Diplopoda: Polydesmida) and the gene order of the two flat-backed millipedes is novel among known myriapod mitochondrial genomes. Unique translocations have occurred, including inversion of one half of the two genomes with respect to other millipede genomes. Inversion of the entire side of a genome (*trnF-nad5-trnH-nad4-nad4L*, *trnP*, *nad1-trnL2-trnL1-rrnL-trnV-rrnS*, *trnQ*, *trnC* and *trnY*) could constitute a common event in the order Polydesmida. Last, our phylogenetic analyses recovered the monophyletic Progoneata, subphylum Myriapoda and four internal classes.

## Introduction

The Myriapoda comprise more than 18,000 species worldwide and are a diverse and ecologically important group of terrestrial arthropods (Miyazawa et al. 2014). Four groups have been united as Myriapoda: the Chilopoda (centipedes) and Diplopoda (millipedes), containing the vast majority of species, and the poorly investigated Pauropoda and Symphyla. An ancient group, the myriapods inhabited Pangea, and as a result they occur on all continents today except for Antarctica (Edgecombe 2004, 2010). Diplopoda (millipedes) is the third most diverse class of Arthropoda, with more than 11,000 species described and an estimated diversity of 80,000 species (Sierwald and Bond 2007; Shelley 2007; Zhang 2011; Brewer et al. 2012; Enghoff et al. 2015). Millipedes are an important component of modern terrestrial ecosystems and play a major role in the breakdown of organic matter (Hopkin and Read 1992). However, there are few studies documenting aspects of the group’s phylogeny, evolution, behavior, physiology, and ecology (Hoffman et al. 2002).

Extant myriapods have not been the subject of extensive molecular phylogenetic study and studies that have been done tend to focus on relationships at the phylum level. Comparative morphological, molecular and higher-level systematic evidence has largely confirmed the monophyly of myriapods (Boudreaux 1979; Ax 1999; Bitsch and Bitsch 2004; Gai et al. 2006; Bäcker et al. 2008; Regier et al. 2008, 2010; Rehm et al. 2014; Fernández et al. 2016), even though this was once a controversial topic (Pocock 1893; Snodgrass 1952; Dohle 1980). The phylogeny of the millipedes is still an open topic regarding their position within the Myriapoda and earliest splits inside the diplopod lineage (Edgecombe 2011; Fernández et al. 2016).

Mitochondrial genomes are used extensively to study phylogeography and phylogenetic relationships (Boore et al. 1998; Gissi et al. 2008; Cameron 2014). In addition to the sequences of mitochondrial genes, the secondary structures of RNAs as well as the mitochondrial gene order have been explored in a phylogenetic context (Boore 1999; Carapelli et al. 2004; Chen et al. 2011; Song et al. 2016). As in most other arthropods, myriapod mitochondrial (mt) genomes are a single circular DNA molecule encoding 13 proteins, 22 transfer RNAs (tRNAs), two ribosomal RNAs (rRNAs), and one A+T-rich region involved in the control of mtDNA replication and transcription.

Gene order in the centipede *Cermatobius longicornis* and the millipede *Prionobelum* sp. is identical to that of *Limulus polyphemus* (Arthropoda: Xiphosura) (Lavrov et al. 2000a; Dong et al. 2012b; Brewer et al. 2013; Gai et al. 2013). However, the arrangement of genes in mt genomes is remarkably variable in *Strigamia maritima* (Chilopoda: Geophilomorpha) and *Symphylella* sp. (Symphyla: Scolopendrellidae) (Gai et al. 2008; Robertson et al. 2015). All millipedes in which the mt genome has been sequenced, except Sphaerotheriida, have *nad6* + *cob* placements that differ from that of *Limulus Polyphemus* and the *nad6* + *cob* pattern was supposed to be sound molecular evidence supporting the Helminthomorpha clade. Dong et al. (2012a) compared nine known myriapod mt genomes and posited that a translocation of *trnT* out of the 5' end of *nad4L* is a common event in derived progoneate lineages. Although taxon sampling is limited, gene synteny has supplied evolutionary evidence relating to Myriapoda phylogenetic and evolutionary history. Full mitochondrial genomes of sixteen myriapod species have hitherto been sequenced, however, this number is still far from sufficient considering the high species richness of this group (Sierwald and Bond 2007; Budd and Telford 2009; Dong et al. 2012a; Robertson et al. 2015). This lack of mitochondrial genome data is hampering phylogenetic and evolutionary studies within the subphylum Myriapoda.

Compositional heterogeneity and accelerated substitution rates have proven to be major sources of systematic bias in mtDNA based phylogeny (Rota-Stabelli et al. 2011). Avoiding inadequate outgroups, selecting conserved amino acid alignment regions and bolstering taxon sampling are keys to phylogenetic reconstruction using mt genomes (Rota-Stabelli et al. 2011; Chen et al. 2014; Robertson et al. 2015). However, complete representation of the four myriapod classes in many studies is not included (Rota-Stabelli et al. 2011; Brewer et al. 2013) and the mt genomes of class Pauropoda, the presumed sister lineage of millipedes, are available in Genbank but not included in previous studies (Brewer et al. 2013). Relationships among the Myriapoda remain unresolved in Robertson et al. (2015), including among the four classes.

Prior to our study, the mt genomes of one flat-backed millipede representing the family Xystodesmidae: *Appalachioria falcifera* (Keeton, 1959) was sequenced. In this species, the entire side of the mt genome is inverted and all genes are on a single strand. Whole genome shotgun reads sequenced with the Illumina sequencing platform were used to obtain two complete mt genomes from the millipedes *Asiomorpha coarctata* and *Xystodesmus* sp. These species are representatives of the families Paradoxosomatidae and Xystodesmidae and further our understanding of how gene rearrangement occurred in the Polydesmida. Phylogenetic analysis including myriapods, three other arthropod classes and outgroups (species of Onychophora and Priapulida) were also performed to explore the internal relationships within the Myriapoda using sequence alignments from mitochondrial genes.

## Methods

### Taxon sampling, DNA extraction and PCR

Specimens of *Asiomorpha coarctata* and *Xystodesmus* sp. were collected from Langya Mountain, Chuzhou, Anhui, China (32°16'N, 118°16'E) and stored at the Molecular Biology Laboratory, Chuzhou University, Chuzhou, Anhui, China (MBLCZU). Species identification was performed by Dong Y (first author) and Qian CY (Shanghai Institutes for Biological Sciences, Chinese Academy of Sciences, Shanghai, China). Voucher specimens (MBLCZU000145, MBLCZU000146) were deposited at the MBLCZU. Total genomic DNA was extracted from one individual representing each species using the DNeasy tissue Kit (Qiagen China, Shanghai).

Standard PCR reactions to amplify three different fragments of mtDNA (*cox1*, *cob* and *nad5*) were undertaken for each sample. Primers are listed in Suppl. material 1: Table S1. Amplified PCR products were gel-purified and then analyzed by primer walking on an ABI-PRISM3730 Automated DNA Sequencer.

### Genome sequencing and analyses

For Illumina sequencing, double index sequencing libraries with average insert sizes of around 300 bp were prepared. The libraries were sequenced as 250 bp paired-end runs on an Illumina Hi-Seq 2000 (about 2 Gb raw data each species). The resulting bait sequences (*cox1*, *cob* and *nad5*) were subsequently employed as references in the manner detailed below. De novo assemblies were conducted with Geneious v8.1 using the Map to Reference program with the following settings applied: medium-low sensitivity/fast; iterate up to five times; with a maximum of 2% mismatches, a maximum gap size of 3 bp and requiring a minimum overlap of 100 bp; do not trim. After manual inspection, the longest contigs resulting from the respective assemblies were then aligned and ensured for correct translation frames with MEGA v5.0, together with reference protein-coding gene sequences from seven millipedes (*Narceus annularus*, *Thyropygus* sp., *Antrokoreana gracilipes*, *Appalachioria falcifera*, *Abacion magnum*, *Brachycybe lecontii*, *Prionobelum* sp.). The contig ends of *Asiomorpha coarctata* and *Xystodesmus* sp. overlapped.

### Mitochondrial genome annotation and analyses

The assembled consensus sequence of each millipede mtDNA was further annotated and analyzed. Preliminary annotation using MITOS webserver provided overall information on mt genomes (Bernt et al. 2013). Protein-coding genes were annotated by identification of their open reading frames, and alignments of homologous genes of other reported myriapod mt genomes. Blast searches in the National Center for Biotechnology Information also helped to identify and annotate the PCGs. Transfer RNA genes were identified by comparing the results predicted by tRNAscan-SE Search Server v.1.21 and ARWEN based on cloverleaf secondary structure information (Lowe and Eddy 1997; Laslett and Canback 2008). Based on known gene order information, the boundaries of the 16S rRNA (rrnS) gene were assumed to be delimited by the ends of the *trnV* and *trnL2* pair. The 12S rRNA (*rrnL*) gene was assumed to start from the end of *trnV*, and its end was roughly identified by alignment with other published millipede sequences. Nucleotide frequencies and codon usage were determined by MEGA v5.05 (Tamura et al. 2007).

### Data sets and sequence alignment

Phylogenetic trees were reconstructed focused on the Arthropoda defining Onychophora and Priapulida as outgroups in the analyses, including 31 taxa (Suppl. material 1: Table S2). Amino acid sequences of 13 mitochondrial protein-coding genes were aligned separately using Clustal X 1.81 based on default settings for each gene (Thompson et al. 1997) then used as guides to align nucleotide sequences. GenBank accession numbers for taxa used in this study are given in Suppl. material 1: Table S1. Each alignment was analyzed with Gblocks under default settings to select conserved amino acid aligned regions (Castresana 2000).

### Phylogenetic analyses

The optimal partition strategy and models were selected by PartitionFinder v1.1.1. We created an input configuration file that contained 13 pre-define partitions by gene. We used the ‘greedy’ algorithm with branch lengths estimated as ‘unlinked’ and Akaike Information Criterion (AIC) to search for the best-fit scheme (Suppl. material 1: Table S3). Model selection of the amino acid data set was performed with ProtTest version 2.4 (Abascal et al. 2007), and under AIC, MtRev+I+G was the best-fit model.

Maximum likelihood phylogenetic analysis searches were carried out with RAxML through a web portal (http://phylobench.vital-it.ch/raxml-bb/index.php). Bootstrap values, indicating the robustness of the internal nodes of the gene trees, were set at 100 replicates. Bayesian analyses of nucleotide and amino acid data sets were performed with MrBayes v3.1.2 (Ronquist and Huelsenbeck 2003), using the GTR+I+G model and MtRev+I+G model, respectively. Four Markov chains were run for 2×10^6^ generations and sampled every 100 generations to yield a posterior probability distribution of 2×10^4^ trees. The first 2000 trees were discarded as burn-in. Three replicates of these Bayesian runs were conducted, retrieving the same topology.

## Results

### Mitochondrial genome organization

The complete mt genome of *Asiomorpha coarctata* (GenBank accession KU721885) is 15,644 bp long and was assembled with coverage between 54–1062 reads; the genome of *Xystodesmus* sp. (GenBank accession KU721886) is 15,791 bp in length and has a coverage within 86–1392 reads. The sizes of these two mt genomes are within the range reported for those of other myriapods ranging from 14,487–16,833 bp (GenBank accessions NC_016676 and NC_021403).

**Figure 1.**
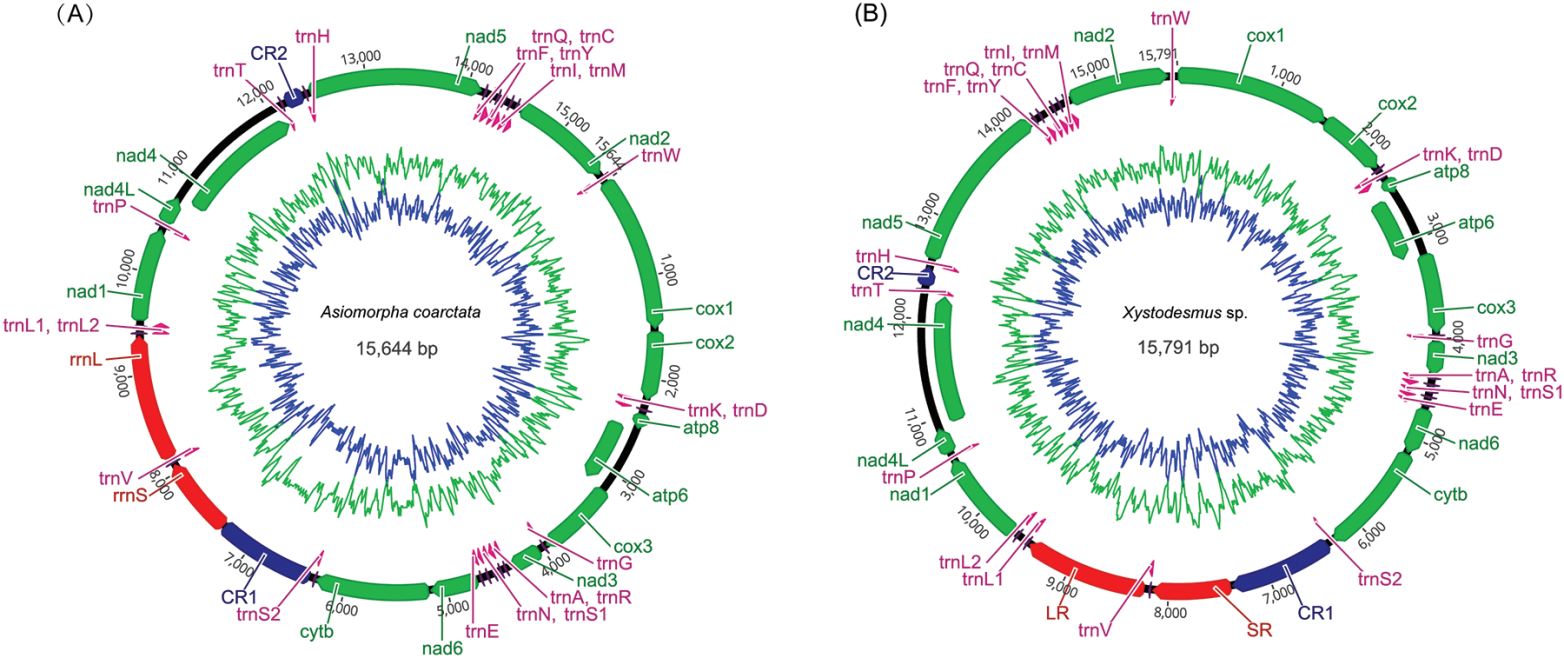
Mitochondrial genomes of the two millipedes sequenced in this study. **A** *Asiomorpha coarctata* **B** *Xystodesmus* sp. Circular maps were drawn with Geneious v9.1.2. Arrows indicate the orientation of gene transcription. Abbreviations of gene names are: *atp6* and *atp8* for ATP synthase subunits 6 and 8; *cox1*–*3* for cytochrome oxidase subunits 1–3; *cob* for cytochrome b, *nad1*–*6* and nad4L for NADH dehydrogenase subunits 1–6 and 4L; and lrRNA and srRNA for large and small rRNA subunits. tRNA genes are indicated with their one-letter corresponding amino acids. CR for control region. The GC content was plotted using a green sliding window and the AT content was blue.

The two genomes encode 13 protein-coding regions, 22 tRNA and two rRNA genes, consistent with metazoan mitochondrial DNA structure, but contain a number of unique features (Figure 1). Most protein-coding genes start with the codon ATG, with the exception of *nad1*, *nad2*, *nad3*, and *nad4*, which begins with TTG, TTG, GTG, and GTG, respectively. Seven protein-coding genes use the typical termination codons TAG (*cox1*, *cox2*, *nad4L*, *nad4* and *nad5*) and TAA (*atp8* and *nad1*), while others in the mt genome of *Asiomorpha coarctata* use incomplete stop codons (Table 1). Several genes show complete stop codons, either TAG (as in *cox1*, *cox2*, *nad3*, *nad4L* and *nad5*) or TAG (as in *atp8* and *cytb*) in *Xystodesmus* sp. (Table 2). Incomplete stop codons are frequently found in other myriapod mitochondrial protein-encoding genes (Dong et al. 2012a) and may be completed by polyadenylation after cleavage of the polycistronic transcript (Ojala et al. 1981). Both novel genomes have an overlapping gene region only between *atp8*/*atp6*.

**Table 1.** Organization of the mitochondrial genome of *Asiomorpha coarctata*.

Feature	From	To	Length (nt)	Codons		Spacer/Overlap(-)
				Start Stop		
*cox*1	1	1533	1533	ATG	TAG	11
*cox2*	1545	2222	678	ATG	TAG	8
*trnK*	2231	2297	67	CTT		1
*trnD*	2299	2363	65	GTC		0
*atp8*	2364	2522	159	ATG	TAA	-7
*atp6*	2516	3182	667	ATG	TA	0
*cox3*	3183	3967	785	ATG	TA	0
*trnG*	3968	4031	64	TCC		0
*nad3*	4032	4381	334	GTG	TA	0
*trnA*	4382	4443	62	TGC		0
*trnR*	4444	4509	66	TCG		2
*trnN*	4512	4579	68	GTT		0
*trnS1*	4580	4638	69	GCT		0
*trnE*	4639	4703	65	TTC		0
*nad6*	4704	5176	473	ATG	AGT	0
*cytb*	5177	6297	1121	ATG	TA	0
*trnS2*	6298	6358	61	TGA		0
CR1	6359	7321	963			0
*rrnS*	7322	8097	776			0
*trnV*	8098	8162	65	TAC		0
*rrnL*	8163	9402	1240			0
*trnL1*	9403	9465	63	TAG		0
*trnL2*	9469	9533	65	TAA		3
*nad1*	9534	10460		TTG	TAA	0
*trnP*	10461	10524	64	CCA		0
*nad4L*	10525	10806	62	ATG	TAG	0
*nad4*	10800	12143	1344	GTG	TAG	0
*trnT*	12144	12206	63	TGT		0
*CR2*	12207	12393	187			0
*trnH*	12394	12458	65	GTG		1
*nad5*	12460	14160	1701	ATG	TAG	3
*trnF*	14164	14231	68	GAA		0
*trnY*	14232	14299	68	GTA		1
*trnQ*	14301	14371	71	TTG		0
*trnC*	14372	14436	65	GCA		0
*trnI*	14437	14503	67	GAT		0
*trnM*	14504	14570	67	CAT		0
*nad2*	14571	15575	1005	TTG	GGA	0
*trnW*	15576	15644	69	TCA		0
Total		15644				-7/30

**Table 2.** Organization of the mitochondrial genome of *Xystodesmus* sp.

Feature	From	To	Length (nt)	Codons		Spacer/Overlap(-)
				Start Stop		
*cox1*	1	1533	1533	ATG	TAG	6
*cox2*	1540	2217	678	ATG	TAG	3
*trnK*	2221	2287	67	CTT		0
*trnD*	2288	2351	64	GTC		0
*atp8*	2352	2513	162	ATG	TAA	-7
*atp6*	2507	3175	669	ATG	TA	0
*cox3*	3179	3963	785	ATG	TA	0
*trnG*	3964	4029	66	TCC		0
*nad3*	4030	4380	351	GTG	TAG	1
*trnA*	4382	4445	64	TGC		4
*trnR*	4450	4514	65	TCG		3
*trnN*	4518	4579	68	GTT		0
*trnS1*	4580	4640	61	GCT		2
*trnE*	4643	4710	68	TTC		2
*nad6*	4713	5181	469	ATG	T	0
*cytb*	5182	6303	1122	ATG	TAA	0
*trnS2*	6304	6360	57	TGA		0
CR1	6361	7392	963			0
*rrnS*	7393	8172	780			0
*trnV*	8173	8238	66	TAC		0
*rrnL*	8239	9465	1227			0
*trnL1*	9466	9530	65	TAG		51
*trnL2*	9582	9650	69	TAA		3
*nad1*	9651	10575	925	TTG	T	0
*trnP*	10576	10640	65	TGG		2
*nad4L*	10643	10924	282	ATG	TAG	0
*nad4*	10918	12264	1347	GTG	TAA	1
*trnT*	12266	12327	62	TGT		0
*CR2*	12328	12535	208			0
*trnH*	12536	12599	64	GTG		1
*nad5*	12602	14299	1698	ATG	TAG	3
*trnF*	14303	14369	67	GAA		0
*trnY*	14370	14434	65	GAT		15
*trnQ*	14450	14515	66	TTG		2
*trnC*	14518	14582	65	GCA		3
*trnI*	14586	14650	65	GAT		0
*trnM*	14651	14716	66	CAT		0
*nad2*	14717	15722	1006	TTG	T	0
*trnW*	15723	15790	68	TCA		1
Total		15791				-7/103

### Non-coding regions

Some millipede mt genomes that have been sequenced (e.g. *Antrokoreana gracilipes*) include two major non-coding regions and others contain a single non-coding region, such as *Narceus annularus*, *Prionobelum* sp. and *Appalachioria falcifera* (Figure 5). Of the genomes sequenced here, *Asiomorpha coarctata* and *Xystodesmus* sp. include two major non-coding regions (Table 1 and 2).

**Figure 2.**
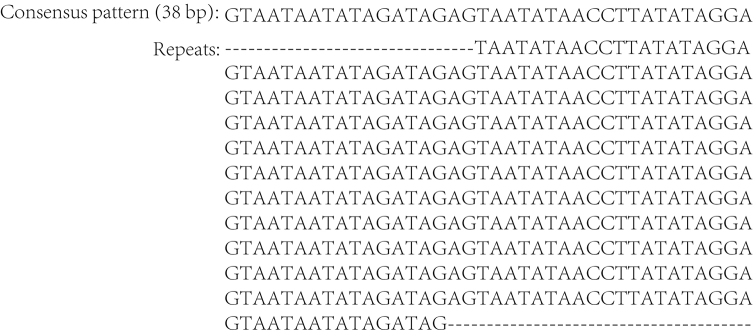
Sequences of the non-coding region in *Asiomorpha coarctata*, primary structures of tandemly repeated regions (11.4 × 38 bp).

The largest non-coding region (CR1, 963 bp) is located between *trnS2* and *rrnS* in *Asiomorpha coarctata* (Table 1). The non-coding region in *Asiomorpha coarctata* contains tandemly repeated regions (11.4 × 38 bp), and the repeated unit is ‘GTAATAATATAGATAGAGTAATATAACCTTATATAGGA’ (Figure 2).

### Transfer RNA

**Figure 3.**
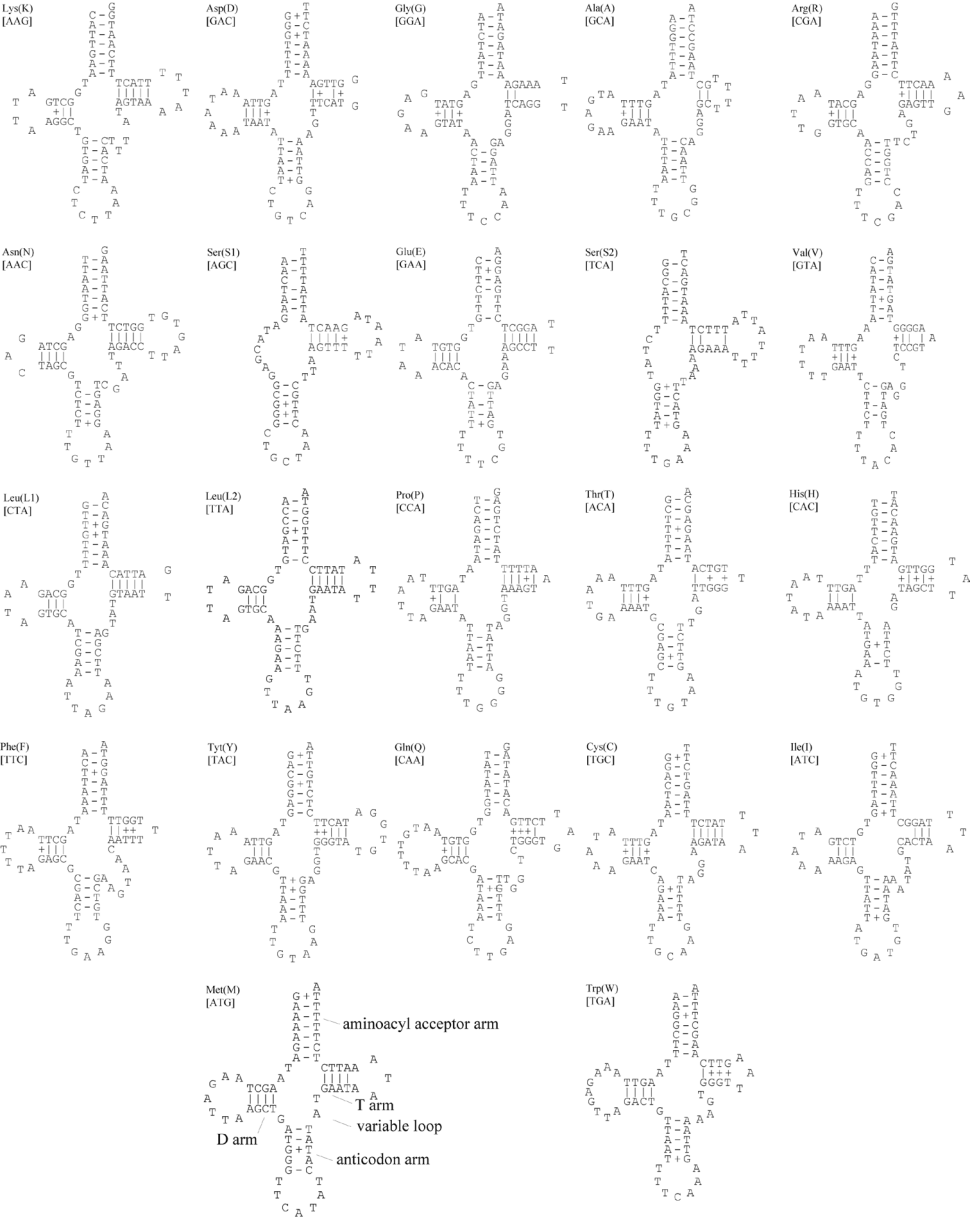
Putative secondary structures of the 22 tRNA genes of *Asiomorpha coarctata*. Watson-Crick base-pairing is indicated by solid lines, and G–T pairs are indicated with plus signs.

**Figure 4.**
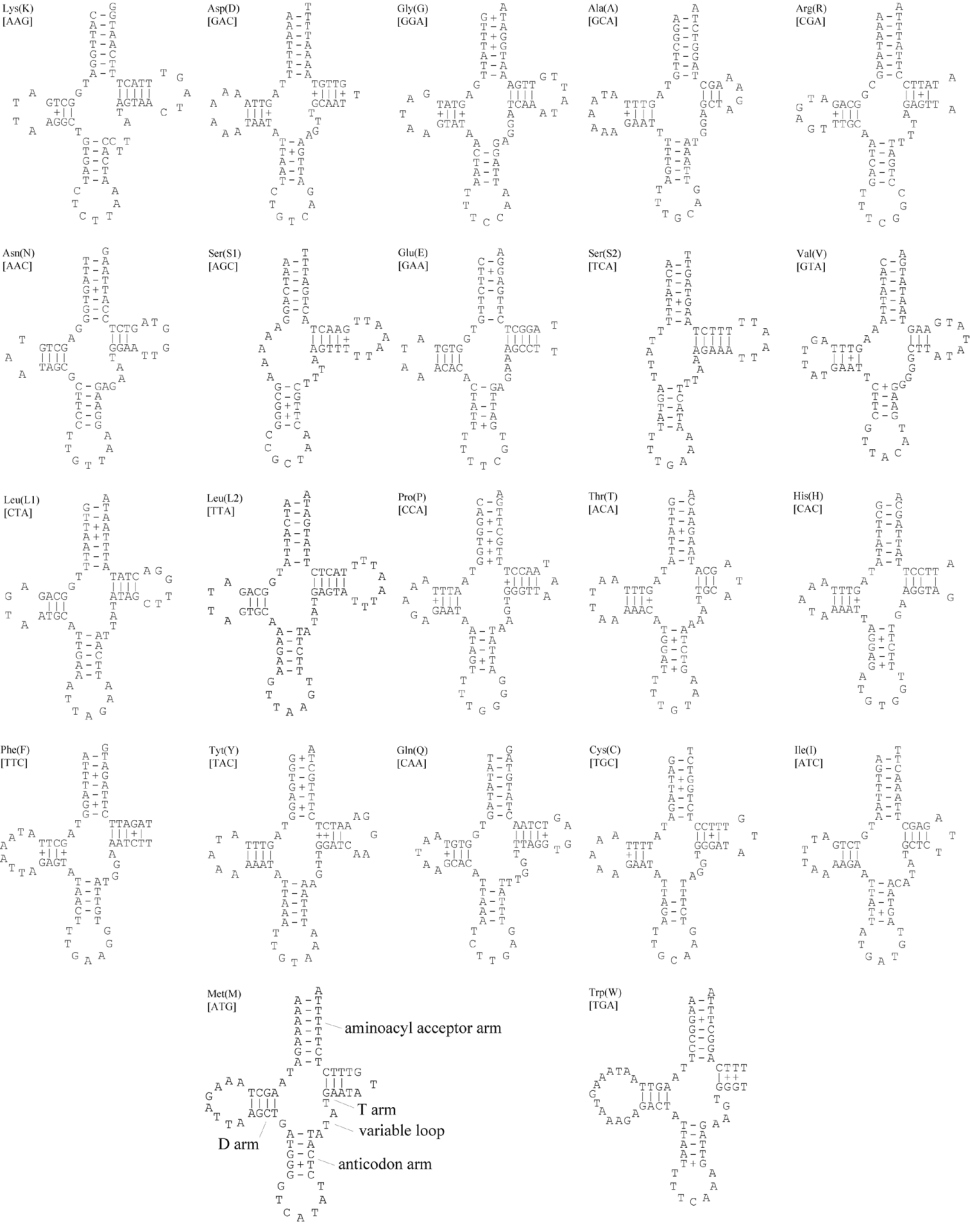
Putative secondary structures of the 22 tRNA genes of *Xystodesmus* sp. Watson-Crick base-pairing is indicated by solid lines, and G–T pairs are indicated with plus signs.

There are 22 potential tRNA genes in *Asiomorpha coarctata* and *Xystodesmus* sp., respectively (Figure 3 and 4), as there are in most other published metazoan mtDNAs (Gissi et al. 2008). All of these tRNA genes are α-strand-encoded bearing more protein-coding sequence (Figure 1), and the newly sequenced mt genomes show dihydrouridine arm (DHU arm, D-arm) loss in *trnS1* and *trnS2*. According to our analysis based on the ARWEN program, *trnS1* lacks the D-arm in all other millipede species.

### Gene order

**Figure 5.**
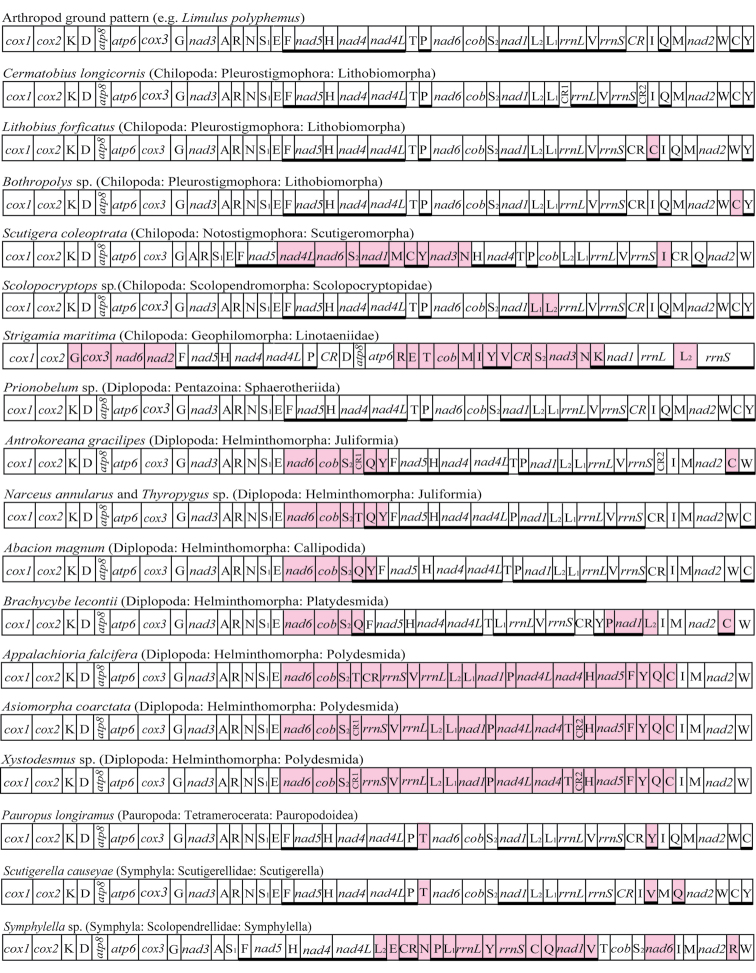
Comparison of gene arrangements in mtDNA of the arthropod ground pattern. Gene segments are not drawn to scale. Genes shaded gray have different relative positions compared to the ground pattern. Underlining indicates the gene is encoded on the opposite strand, and arrows indicate translocation of *trnT*. CR: putative control region. Gene arrangements of two diplopods, *Narceus annularus* and *Thyropygus* sp. are similar and represented as one.

Gene order arrangements were compared with mt genome organization in other myriapods (Figure 5), including the ancestral gene order of the mitogenome for the Myriapoda shared by *Prionobelum* sp. and *Limulus polyphemus* (Dong et al. 2012b). The overall arrangement of the genes around the *Asiomorpha coarctata* and *Xystodesmus* sp. mt genomes is unique compared to other myriapod species. All coding regions are on a single strand in *Asiomorpha coarctata* and *Xystodesmus* sp. which has been reported in the mt genome of the millipede *Appalachioria falcifera* with an entire side of the genome inverted (Brewer et al. 2013). These three flat-backed millipedes are identical but for a single tRNA translocation in *Asiomorpha coarctata* and *Xystodesmus* sp. The two newly sequenced mt genomes have undergone gene and tRNA translocations compared to other myriapod sequences.

### Phylogenetic inference

**Figure 6.**
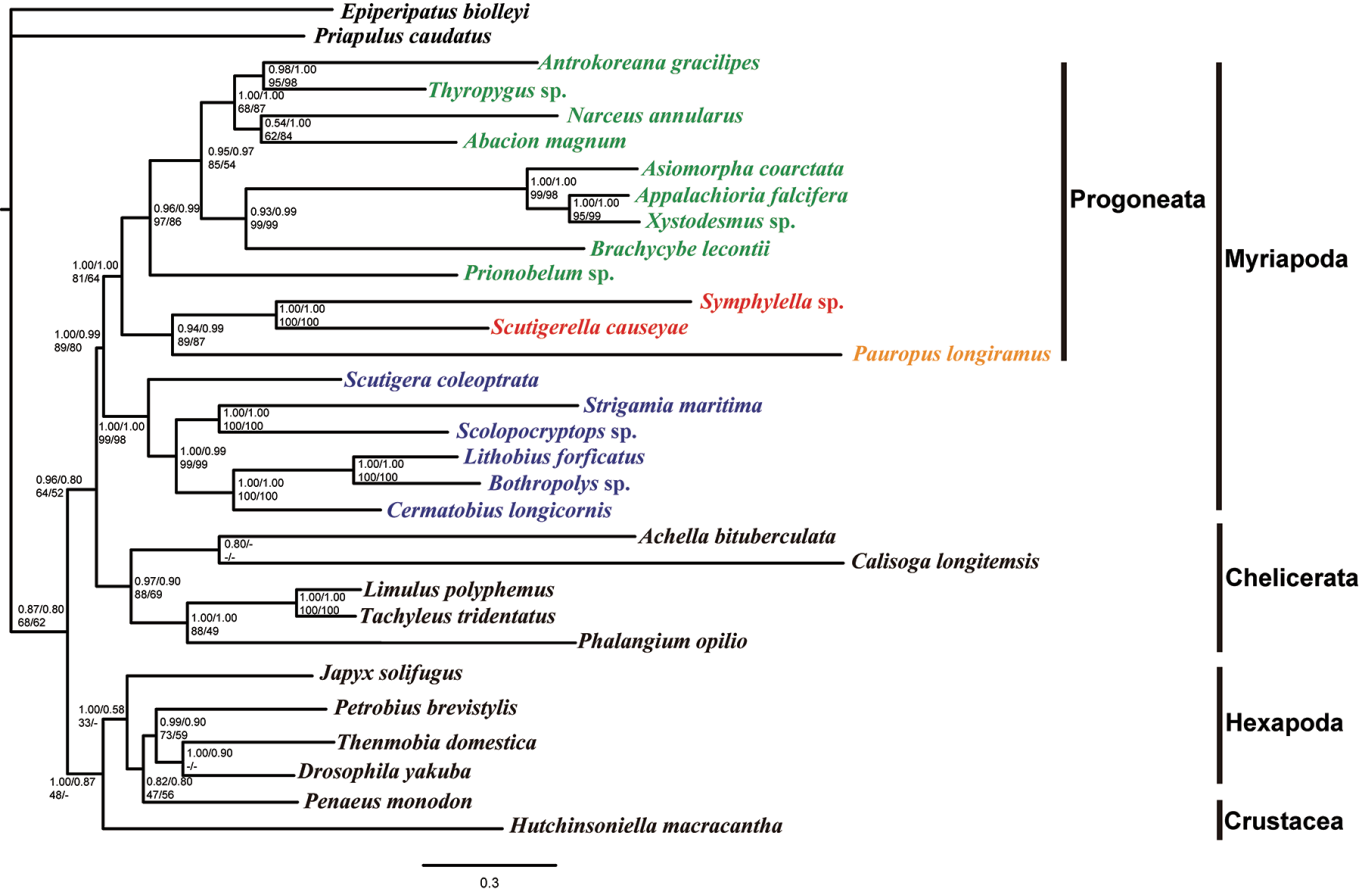
Phylogenetic tree of the Arthropoda, including Myriapoda, Hexapoda, Crustacea and Chelicerata and outgroups reconstructed based on protein-coding genes from mtDNA genomes. Each group of four numbers indicates node confidence values (from top left): Bayesian posterior probabilities in percent (BPP) in amino acid and nucleotide datasets; maximum likelihood bootstrapping values (MLBP) in amino acid and nucleotide datasets.

Bayesian inference and maximum likelihood phylogenetic analysis were performed using conserved blocks of amino acid and nucleotide data sets (Figure 6). The topological pattern obtained using Bayesian inference and maximum likelihood analyses based on both the amino acid and nucleotide data sets were similar.

Within the Diplopoda, the Polydesmida clade, including *Appalachioria falcifera*, *Asiomorpha coarctata*, and *Xystodesmus* sp. is well supported. A clade consisting of Polydesmida plus Colobognatha (*Brachycybe lecontii*) is also recovered. All millipedes in this study except *Prionobelum* sp. recovered as a monophyletic group, Helminthomorpha. The grouping with Helminthomorpha and Pentazonia (the basal lineage *Prionobelum* sp.) is supported.

Four myriapod clades are resolved, with Chilopoda (BPP = 1.00 and 1.00; MLBP = 99 and 98) as the basal group. A monophyletic Progoneata (“Symphyla + Pauropoda” + Diplopoda) is recovered as the sister group of the Chilopoda (BPP = 1.00 and 0.99; MLBP = 89 and 80). Our Bayesian inference analysis recognizes a monophyletic Myriapoda with strong support, while the maximum likelihood analysis is only weakly supported.

## Discussion

Next-generation sequencing such as the 454 pyrosequencing, Solexa, and SOLiD provided by Roche, Illumina and Applied Biosystems, has the ability to generate a large number of sequences within a very short time compared to Sanger’s method (Chilana et al. 2012). These methods have been fused to rapid generate sequence data and successful de novo sequence assemblies for arthropods (Kirkness et al. 2010) and more recently to the assembly of full mt genomes (Knaus et al. 2011; Ma et al. 2012; Coates 2014; Aguado et al. 2016). Next-generation sequencing results in better assembly, leading to fewer gaps, larger contigs and greater accuracy of the final consensus sequence. It is essential for accurately identifying more complex rearrangements, for example in the order Polydesmida, which has a large inversion in the mt genome in this study.

We found that the genome size was larger in *Asiomorpha coarctata* (15,644 bp) and *Xystodesmus* sp. (15,791 bp) than other millipede mt genomes (14747–15282 bp; *Antrokoreana gracilipes*, GenBank accession NC_010221; *Appalachioria falcifera*, GenBank accession NC_021933). Intergenic spacer length variation may have arisen through retention of partial duplication, or incomplete multiple deletions of redundant genes (McKnight and Shaffer 1997; Yamauchi et al. 2003) under the duplication-and-deletion mechanism. For this reason, we speculate that multiple intergenic spacers distributed in the two larger mt genomes may serve as a guide in deducing derived gene arrangement.

Most of the tRNAs appear to be truncated and lack one of the arms found in the canonical tRNAs of pauropods and symphylans (Gai et al. 2008; Dong et al. 2012a). One to two nucleotide mismatches could be found in the acceptor arms of the five tRNAs in *Asiomorpha coarctata*, and in the four tRNAs in *Xystodesmus* sp. Nucleotide mismatch in the arms of tRNAs may also occur in other myriapod groups (Woo et al. 2007; Lavrov et al. 2000b; Gai et al. 2008, 2013; Brewer et al. 2012; Dong et al. 2012a, 2012b).

Compared with other millipedes, the mt gene order in *Asiomorpha coarctata* and *Xystodesmus* sp. is very similar to that in *Appalachioria falcifera*. These three species belong to the order Polydesmida (Figure 5). There are no differences in the relative position of the protein-coding genes, but the *trnT* gene and non-coding regions of *Appalachioria falcifera* mt genome are translocated with respect to the newly sequenced mt genomes here. Although only nine millipede mt genomes are compared, an extreme variety in gene arrangements is known in millipedes, and inversion of an entire side of the genome (*trnF-nad5-trnH-nad4-nad4L*, *trnP*, *nad1-trnL2-trnL1-rrnL-trnV-rrnS*, *trnQ*, *trnC and trnY*) could be a synapomorphy in the Polydesmida lineage.

Gene arrangement in three flat-backed millipedes is similar to that in other Helminthomorpha sequenced previously in which *nad6* + *cob* placements occurred. We agree with Brewer et al. (2013) that the inversion of the mt genome in flat-backed millipedes is a derived event associated with losing the second non-coding region. The mitochondrial gene arrangements of the order Polydesmida and the infraclass Helminthomorpha lineages are reshuffled regularly. To better understand the evolutionary implications of gene arrangements in the Myriapoda, mt genome research with broader taxon sampling will be required.

This phylogenomic study provides a strongly supported phylogenetic framework for the monophyletic origin of the Myriapoda and the monophyly of the Progoneata and extant myriapod subgroups. Among the Myriapoda, the union of Diplopoda with Pauropoda as a monophyletic group (= Dignatha) was once widely accepted (Dohle 1980, 1998; Enghoff et al. 1993; Blanke and Wesener 2014; Edgecombe 2015), but a number of molecular analyses of nuclear and mitochondrial sequences support the combination of Pauropoda and Symphyla (Gai et al. 2006; Regier et al. 2010; Dong et al. 2012a). Our results strongly support the traditional morphology-based Progoneata (Diplopoda + Pauropoda + Symphyla) defined by the presence of gonopores behind the second pair of legs. The notion of Progoneata recovered here is consistent with that favored by morphology and molecular analyses (Gai et al. 2008; Edgecombe 2010, 2011; Regier et al. 2010; Dong et al. 2012a).

The Polydesmida and Helminthomorpha are recovered as monophyletic. Gene order in *Prionobelum* sp. which is the basal lineage of the millipede mt genome is assumed to represent the millipede or myriapod ground pattern (Dong et al. 2012b), and inversion of the entire side of the genome occurred as a common event in the order Polydesmida lineage proposed in this study. In our gene order comparison of these millipedes, phylogenetic results are mainly concordant with gene arrangement analyses (Figure 5). Combining the implications of phylogenetic analyses and gene arrangement could yield valuable understanding of myriapod evolutionary history.

Polydesmida has been considered the sister group of Juliformia and a ‘ring-forming’ clade from the perspective of morphology (Enghoff et al. 1993). Polydesmida also unite the Nematophora (including Stemmiulida, Callipodida and Chordeumatida) as sister group for the shared presence of preanal spinnerets (Enghoff 1984; Sierwald et al. 2003; Shear 2008; Blanke and Wesener 2014). The Polydesmida allied with Platydesmida (basal representatives of the subclass Colobognatha) as a clade in our phylogenetic analyses. The combination Polydesmida + Colobognatha has been recovered in previous analyses (Sierwald and Bond 2007; Brewer et al. 2013). More taxa must be sequenced from the Colobognatha and better analysis methods used to test the position of the Polydesmida.

Many internal relationships of the Diplopoda remain unresolved and several groups are paraphyletic. The Bayesian inference tree seems to have better support at shallower nodes with amino acid data sets, whereas the maximum likelihood tree has better support at deeper nodes. The maximum likelihood tree has very low support at most nodes and confuses the relationships of taxa that are confidently placed in monophyletic groups by other studies (Meusemann et al. 2010; Regier et al. 2010; Brewer et al. 2013). Our finding that the Bayesian methods outperformed likelihood-based approaches is consistent with the results reported by Talavera and Castresana (2007).

## Supplementary materials

Supplementary material 1 Supplementary tables Data type: molecular data This dataset is made available under the Open Database License (http://opendatacommons.org/licenses/odbl/1.0/). The Open Database License (ODbL) is a license agreement intended to allow users to freely share, modify, and use this Dataset while maintaining this same freedom for others, provided that the original source and author(s) are credited.Yan Dong, Lixin Zhu, Yu Bai, Yongyue Ou, Changbao Wang
