# Supplementary material for: Complete mitochondrial genomes of two flat-backed millipedes by next-generation sequencing (Diplopoda, Polydesmida)
Source: Zookeys. 2016 Nov 28;(637):1–20. doi: 10.3897/zookeys.637.9909 (PMC5240118; doi:10.3897/zookeys.637.9909)
Supplement: Supplementary material 1 — Supplementary tables [file zookeys-637-001-s001.docx]

**Supplementary** Table S1. PCR primer pairs, sequences used in this study.

| Primer name | Nucleotide sequence (5’-3’) |
| --- | --- |
| CO1CF | GCACGTCTACAAATCATAAAGATATTGG |
| CO1CR | TAAACTTCAGGGTGACCGAAAAATCA |
| COBF | GGTTTATTTTTAGCTATACACTACAC |
| COBR | GCAAATAGAAAATATCATTCTGGTTG |
| ND5F | GAATAATAAAGCCTTAAATAAAGCATG |
| ND5R | TTATCTAATCGAATTGGAGATGT |

**Supplementar**y**Table S2**. GenBank accession numbers for taxa used in this study

| Taxon |  | Species | Accession No. |  |
| --- | --- | --- | --- | --- |
| Myriapoda |  |  |  |  |
| Chilopoda |  |  |  |  |
| Lithobiomorpha | Lithobiidae | *Lithobius forficatus* | NC_002629 | Lavrov et al. 2000 |
| Lithobiomorpha | Ethopolyidae | *Bothropolys* sp. | NC_009458 | Park 2004 |
| Lithobiomorpha | Henicopidae | *Cermatobius longicornis* | NC_021403 | Gai et al. 2013 |
| Scutigeromorpha | Scutigeridae | *Scutigera coleoptrata* | NC_005870 | Negrisolo et al. 2004 |
| Scolopendromorpha | Cryptopidae | *Scolopocryptops* sp. | KC200076 | Gai et al. 2013 |
| Geophilomorpha | Linotaeniidae | *Strigamia maritima* | NC_026557 | Robertson et al. 2015 |
| Diplopoda |  |  |  |  |
| Spirobolida | Spirobolidae | *Narceus annularus* | NC_003343 | Lavrov et al. 2002 |
| Spirostreptida | Harpagophoridae | *Thyropygus* sp. | NC_003344 | Lavrov et al. 2002 |
| Julida | Nemasomatidae | *Antrokoreana gracilipes* | NC_010221 | Woo et al. 2007 |
| Polydesmida | Xystodesmidae | *Appalachioria falcifera* | NC_021933 | Brewer et al. 2013 |
| Callipodida | Abacionidae | *Abacion magnum* | NC_021932 | Brewer et al. 2013 |
| Platydesmida | Andrognathidae | *Brachycybe lecontii* | NC_021934 | Brewer et al. 2013 |
| Sphaerotheriida | Zephroniidae | *Prionobelum* sp. | NC_018361 | Dong et al. 2012b |
| Polydesmida | Paradoxosomatidae | *Asiomorpha coarctata* | KU721885 | present study |
| Polydesmida | Xystodesmidae | *Xystodesmus* sp. | KU721886 | present study |
| Symphyla |  |  |  |  |
|  | Scutigerellidae | *Scutigerella causeyae* | NC_008453 | Gai et al. 2008 |
|  | Scolopendrellidae | *Symphylella* sp. | NC_011572 | Podsiadlowski et al. 2007 |
| Pauropoda |  |  |  |  |
| Pauropodidae | Tetramerocerata | *Pauropus longiramus* | NC_016676 | Dong et al. 2012a |
| Chelicerata |  |  |  |  |
|  | Ammotheidae | *Achella bituberculata* | NC_009724 | Parks et al. 2003 |
|  | Araneae | *Calisoga longitemsis* | NC_010780 | Masta and Boore 2008 |
|  | Limulidae | *Limulus polyphemus* | NC_003057 | Lavrov et al. 2000 |
|  | Limulidae | *Tachyleus tridentatus* | NC_012574 | Beak et al. 2014 |
|  | Phalangiidae | *Phalangium opilio* | NC_010766 | Masta and Boore 2008 |
| Crustacea |  |  |  |  |
|  | Penaeoidea | *Penaeus monodon* | NC_002184 | Wilson et al. 2000 |
|  | Hutchinsoniellidae | *Hutchinsoniella macracantha* | NC_005937 | Lavrov et al. 2004 |
| Hexapoda |  |  |  |  |
|  | Machilidae | *Petrobius brevistylis* | NC_007688 | Podsiadlowski 2006 |
|  | Lepismatidae | *Thermobia domestica* | NC_006080 | Cook et al. 2005 |
|  | Drosophilidae | *Drosophila yakuba* | NC_001322 | Clary and Wolstenholme 1985 |
|  | Japygidae | *Japyx solifugus* | NC_007214 | Carapelli et al. 2005 |
| Onychophora |  |  |  |  |
|  | Peripatidae | *Epiperipatus biolleyi* | NC_009082 | Podsiadlowski et al. 2007 |
| Priapulida |  |  |  |  |
|  | Priapulidae | *Priapulus caudatus* | NC_008557 | Webster et al. 2006 |

**SupplementaryTable S3.**The best partitioning scheme selected by PartitionFinder for dataset.

| Data matrices | **Subset Partitions** | **Best Model** |
| --- | --- | --- |
| 4 partitions | P1: (*atp6*, *atp8*, *nad2, nad6*) | GTR+I+G |
|  | P2: (*cox1*) | GTR+I+G |
|  | P3: (*cox2*,*cox3*, *cob, nad3*) | GTR+I+G |
|  | P4: (*nad1, nad4, nad4L, nad5*) | GTR+I+G |
